# Urine creatinine in treatment-naïve HIV subjects in eastern Nigeria

**DOI:** 10.11604/pamj.2016.25.139.10580

**Published:** 2016-11-04

**Authors:** Ernest Ndukaife Anyabolu

**Affiliations:** 1 Division of Nephrology, Department of Medicine, Imo State University Teaching Hospital, Orlu, Nigeria; 2 Division of Nephrology, Federal Medical Centre, Owerri, Nigeria

**Keywords:** HIV, urine creatinine, associated factors, Nigeria

## Abstract

**Introduction:**

Human immunodeficiency virus (HIV) infection is a global healthcare problem. Some diseases and physiological states may be altered in HIV-infected individuals. Our objective was to evaluate urine creatinine and factors that influence urine creatinine in treatment-naïve HIV subjects in Nigeria.

**Methods:**

This was a cross-sectional study involving treatment-naïve HIV subjects in a tertiary hospital in Nigeria. Creatinine in spot and 24-hour urine samples and other relevant investigations were performed. Low urine creatinine or dilute urine was defined as 24-hour urine creatinine (24HUCr) <300mg, normal urine creatinine as 24HUCr 300-3000mg and high urine creatinine or concentrated urine as 24HUCr>3000mg.Theassociation of low urine creatinine and high urine creatinine with potential risk factors was determined.

**Results:**

The mean spot urine creatinine (SUCr) of the treatment-naïve HIV subjects was 137.21± 98.47(mg/dl), minimum value 13.3mg/dl, maximum value 533.3mg/dl and range of values 520.0mg/dl. The mean 24HUCr was 1507±781mg, minimum value 206mg, maximum value 4849mg and range of values 4643mg. Twenty four-hour urine creatinine<300mg was observed in 2(0.5%) subjects, normal 24HUCr 300-3000mgin 349(93.1%) subjects and 24HUCr>3000mg in 24(6.4%) subjects. There was significant association between 24HUCr and serum low density lipoprotein cholesterol (LDL),serum high density lipoprotein cholesterol (HDL). There was high correlation between 24HUCr>3000mg and 24-hour urine osmolality (24HUOsm) (r=0.95), body mass index (BMI) (r=0.74), CD4 cells count (r=-0.71), serum HDL (r=-0.73).

**Conclusion:**

The prevalence of dilute urine and concentrated urine was low. Twenty-four hour urine osmolality. BMI, CD4 cells count and HDL were strong correlates of high urine creatinine. Lipid abnormalities were common in treatment-naïve HIV subjects with high urine creatinine. There is need for clinicians to routinely conduct urine creatinine and further search for abnormalities of serum lipids, weight changes, depressed immunity and anemia in HIV subjects with dilute or concentrated urine in the early stages of the infection.

## Introduction

Human immunodeficiency virus (HIV) infection is a global healthcare problem. Sub-Saharan African countries account for about 70% of global HIV-infected individuals.[1] Nigeria has an HIV prevalence of 3.7% [1]. Virtually all organs of the body have been found to be affected directly or indirectly by HIV infection [2]. Similarly, some physiological responses may be altered in persons with HIV infection [3–5]. Creatinine is produced by the muscles, degraded in the liver and excreted by the kidney at a constant rate, influenced by age, gender and weight [6]. Many environmental, physiologic and disease conditions may impact on 24-hour urine creatinine excretion. Excretion of creatinine is also influenced by exogenous substances like cocaine, heavy metals like arsenic and cadmium found in the bio-environment as part of environmental pollutants, meat consumption and medications like cimetidine and trimethoprim. As a result, urine creatinine has been used in monitoring substance use and bioenvironmental pollutants [7–9]. Daily urine creatinine excretion varies over a wide range of values in normal healthy state [10]. Impaired renal function usually results in poor renal secretion of creatinine in the urine; urine creatinine decreases as renal function impairment increases [11]. Studies have identified some associated factors of high 24HUCr. They included age, sex, race, body mass index (BMI), hypertension, water intake, and blood osmolality [12]. On the other hand, low 24HUCr has been found to be associated with glomerular filtration rate, an older age, diabetes, and lower levels of BMI, proteinuria, and protein intake [11]. Another important use of urine creatinine is for evaluating adequacy of 24-hour urine sample collection [13]. Studies are sparse on urine creatinine in HIV subjects emanating from Nigeria. We have, therefore, set out to evaluate urine creatinine and factors which influence low and high urine creatinine in this group of subjects.

## Methods

This was a cross-sectional study, comprising of 375 treatment-naïve HIV-positive subjects, recruited from an HIV clinic of Federal Medical Centre (FMC), Owerri, Nigeria. The study was conducted from April to August 2011. Inclusion criterion was treatment-naïve, HIV-positive subjects within the age range of 16-65 years. Subjects who had known pituitary, adrenal, renal diseases or terminal illness, those who were pregnant were excluded from the study. The study was approved by the Ethical Research Committee of the hospital. The Ethics Approval Reference Number was FMC/HCS/VOL II, dated 16th march, 2011.Informed written consent was obtained from all the subjects that participated in the study. With the aid of a questionnaire, demographic, anthropometric and other relevant data were obtained from the subjects. The purpose of the study was explained to the subjects. The age, gender, place of origin and domicile were obtained. Height was measured and recorded in meter (m). Using a weighing scale, weight was measured and recorded in kilogram (kg). BMI was taken as the ratio of weight/height^2^ (km/m^2^).

Clear instructions were given to all the subjects on how to collect 24-hour urine sample. A day-time random spot urine sample and blood samples were collected at the end of the 24-hour urine sample collection. From the random spot urine samples collected, spot urine protein (SUP), spot urine creatinine (SUCr) and spot urine osmolality (SUOsm) were performed. Also from the 24-hour urine samples collected, 24-hour urine protein (24HUP), 24-hour urine creatinine (24HUCr) and 24-hour urine osmolality (24HUOsm) were performed. Serum creatinine was performed on the blood samples collected. Osmolality was determined by freezing point depression method using Precision Osmette 5002 osmometer, creatinine by modified Jeff’s method and protein by photometric method. Creatinine Clearance (ClCr), spot urine creatinine/osmolality ratio (SUCOR) were determined. Other investigations performed included HIV screening and confirmatory tests, CD4 cells count, fasting serum lipid profile (FSLP) {total cholesterol, triglyceride, high density lipoprotein cholesterol (HDL), low density lipoprotein cholesterol (LDL)}, and hemoglobin (Hb).

The potential associated factors of dilute and concentrated urine in treatment-naïve HIV subjects evaluated were CD4 cells, SUOsm, SUP, 24HUP, 24HUOsm, SUCOR, serum cholesterol, serum triglyceride, serum HDL, serum LDL and ClCr.

### Statistical analysis

The data were analyzed using SPSS version 17.0 (SPSS Int. Chicago, II, USA). The distribution and characterization of clinical and laboratory features among HIV-positive subjects with different levels of 24HUCr were analyzed using cross-tabulation, while statistical significance of association of these variables with 24HUCr changes was determined using student t-test. Correlation statistics were used to determine the association of variables with high urine creatinine on one hand and with low urine creatinine on the other hand. Multivariate linear regression analyses were used to determine the strength of variables to predict low urine creatinine and high urine creatinine. P?0.05 was taken as statistically significant.

### Definition of terms

Normal urine creatinine: 24HUCr 300 – 3000mg. Low urine creatinine or dilute urine: 24HUCr <300mg. High urine creatinine or concentrated urine: 24HUCr >3000mg.

## Results

Three hundred and ninety-three treatment-naïve subjects were studied. Eighteen of them were excluded from the study on account of incomplete sample collection. The mean age of the subjects was 39 ± 11 years. For all the HIV participants, the mean spot urine creatinine (SUCr) was 137.21± 98.47(mg/dl), minimum value 13.3mg/dl, maximum value 533.3mg/dl and range of values 520.0mg/dl. The mean 24HUCrwas1507±781mg, minimum value 206mg, maximum value 4849mg and range of values 4643mg (Table 1). Two (0.5%) of the HIV subjects have 24HUCr <300mg, 349(93.1%) have 24HUCr 300 – 3000mgwhile 24(6.4%) have 24HUCr >3000mg. Serum LDL was significantly associated with 24HUCr (p=0.001) in the treatment-naïve HIV subjects. Two subjects have 24HUCr <300mg and both of them have borderline serum LDL. Twenty-four subjects have high urine creatinine, and all of them have desirable serum LDL (Table 2). There was significant association between serum HDL and 24HUCr, p=0.028in the treatment-naïve HIV subjects. Two subjects have 24HUCr<300mg and both have desirable serum HDL<1mg/dl. Twenty-four subjects have 24HUCr>3000mg.Out of these, 83.3% have high serum HDL, while 16.7% have desirable serum HDL. This showed that the prevalence of high urine creatinine increased as serum HDL increased (Table 2). There was no significant association between 24HUCr and BMI, p=0.191, serum total cholesterol, p=0.659, ClCr, p=0.265, 24HUP, p=0.237, CD4 cells count, p=0.677, serum triglyceride, p=0.790, Hb, p=0.140 in the treatment-naïve HIV subjects (Table 2). Significant correlation was obtained between 24HUCr and SUCr (p=019), 24HUV (p=0.004), SUCOR (<0.001), serum LDL (p=0.31), ClCr (p<0.001), serum creatinine (<0.001) in the treatment-naïve HIV subjects. Hemoglobin, SUP, SUOsm, 24HUP, 24HUOsm, serum cholesterol, serum HDL and serum triglyceride did not have significant correlation with 24HUCr (Table 3). There was very strong correlation between 24HUCr>3000mg and 24HUOsm (r=0.95), BMI, (r=0.74), CD4 cells count, (r=-0.71), HDL, (r=-0.73) in the treatment-naïve HIV subjects. However, there was moderate correlation between 24HUCr and 24HUV, (r=0.58), Hb, (r=-0.43). Conversely, there was poor correlation between SUCr and BMI, (r=0.131, p=0.009)), SUP, (r=0.183, p=<0.001), SUOsm, (r=0.288, p=<0.001), 24HUV, (r=-0.111, p=0.032), ClCr, (r=0.108, p=0.036) (Table 4). Multivariate linear regression of 24HUCr >3000mg with its potential risk factors was voided as the colinearity variance was skewed due to the small subpopulation (24), that have 24HUCr >3000mg.

**Table 1:** Characteristics of variables in treatment-naïve HIV subjects

Variables (mean ± SD)	HIV Subjects
Body Mass Index (kg/m^2^)	26.2± 5.4
Hemoglobin (g/dl)	11.2±1.8
CD4 cells/ml (median)	391
SUOsm (mOsm/kgH_2_O)	464±271
Spot Urine Protein(mg/dl)	11.89±19.13
Spot Urine Creatinine (mg/dl)	137.21± 98.47
24-Hour Urine Protein (g)	0.187± 0.290
24-Hour Urine Creatinine (mg)	1507±781
24HUOsm(mOsm/kgH_2_O)	564 ± 501
SUCOR (mg/dl/mOsm/kgH_2_O)	0.422± 0.486
Cholesterol (mmol/l)	4.26± 0.90
Triglyceride (mmol/l)	1.23± 0.37
HDL (mmol/l)	1.18± 0.39
LDL (mmol/l)	2.05 ±0.58
Creatinine Clearance (mls/min)	91.42±22.98

SD=standard deviation, SUOsm=spot urine osmolality, 24UOsm=24-hour urine osmolality, SUCOR=spot urine creatinine/osmolality ratio, HDL=high density lipoprotein cholesterol, LDL=low density lipoprotein cholesterol,

**Table 2:** Distribution and characterization of selected risk factors at different levels of 24-hour urine creatinine in treatment-naïve HIV-positive subjects (n=375)

Variables	24-hour urine creatinine levels (no/%)			Chi square	Likely hood ratio	P value
	<300mg	300-750mg	>750mg			
BMI (kg/m^2^)<18.5	0(0.0%)	22(91.7%)	2((8.3%)	8.702	0.143	0.191
18.5-24.9	0(0.0%)	124(96.9%)	4(3.1%)			
25.0-29.9	2(1.4%)	128(88.9%)	14(9.7%)			
≥30	0(0.0%)	75(94.9%)	4(5.1%)			
CD4 cells count <200	0(0.0%)	41(97.1%)	4(8.9%)	0.781	0.614	0.677
≥200	2(0.6%)	307(93.3%)	20(6.1%)			
Hb (g/dl) ≥12.0	2(1.6%)	108(88.5%)	12(9.8%)	9.644	0.107	0.140
10.0-11.9	0(0.0%)	163(96.4%)	6(3.6%)			
7.0-9.9	0(0.0%)	72(92.3%)	6(7.7%)			
<7.0	0(0.0%)	6(100.0%)	0(0.0%)			
ClCr(mls/min)≥90mls/min	0(0.0%)	183(92.0%)	16(8.0%)	5.229	0.204	0.265
60-89	2(1.4%)	135(94.4%)	6(4.2%)			
30-59	0(0.0%)	31(93.9%)	2(6.1%)			
24HUP<0.300g	2(0.6%)	301(93.8%)	18(5.6%)	8.018	0.178	0.237
≥0.300g	(0.0%)	48(88.9%)	6(11.1%)			
**FSLP (mmol/l)**						
Chol T Des (<5.2)	2(0.6%)	308(92.5%)	23(6.9%)	1.618	0.806	0.659
BorderL (5.2-6.2)	0(0.0%)	35(97.2%)	1(2.8%)			
High (>6.2)	0(0.0%)	6(100.0%)	0(0.0%)			
LDL Des (<2.6)	0(0.0%)	284(92.2%)	24(7.8%)	14.609	<0.001	0.001
BorderL (2.6- 4.1)	2(3.0%)	64(97.0%)	0(0.0%)			
HDL Low (<1)	2(1.5%)	124(95.4%)	4(3.1%)	7.317	0.016	0.028
High (≥1)	0(0.0%)	225(91.8%)	20(8.2%)			
TG Des (<1.7)	2(0.6%)	311(92.3%)	24(7.1%)	3.150	0.449	0.790
BorderL (1.7-2.2)	0(0.0%)	29(100.0%)	0(0.0%)			
High (>2.2)	0(0.0%)	8(100.0%)	0(0.0%)			

LHR=Likelihood ratio, BMI=body mass index, Hb=hemoglobin,ClCr=creatinine clearance, 24HUP=24-hour urine protein, FSLP=fasting serum lipid profile, CholT=total cholesterol, Des=desirable. BorderL=borderline, LDL=low density lipoprotein cholesterol, HDL=high density lipoprotein cholesterol, TG=triglyceride.

**Table 3:** Correlation of 24HUCr with selected variables in treatment-naïve HIV subjects (n=375)

Variables	Correlation coefficient (r)	P value
Body mass index	-0.036.	0.470
Hemoglobin (g/dl)	0.075	0.117
Spot urine protein	0.044	0.538
Spot urine creatinine	0.129	0.019
Spot urine osmolality	0.107	0.058
24-hour urine protein	0.035	0.625
24-hour urine osmolality	0.063	0.167
24-hour urine volume	0.143	0.004
SUCOR	0.288	<0.001
Serum creatinine	0.290	<0.001
Serum cholesterol (total)	0.074	0.242
Serum Triglyceride	-0.075	0.189
Serum HDL	0.029	0.542
Serum LDL	-0.109	0.031
Creatinine clearance	0.367	<0.001

SUCOR=spot urine creatinine osmolality ratio, HDL=high density lipoprotein cholesterol, LDL=low density lipoprotein cholesterol

**Table 4:** Correlation of 24HUCr>3000mg with selected variables in treatment-naïve HIV subjects (n=24)

Variables	Correlation coefficient (r)	P value
Body mass index	0.744	<0.001
Hb (g/dl)	-0.427	<0.001
Spot urine protein	0.397	<0.001
Spot urine creatinine	0.371	<0.001
Spot urine osmolality	-0.549	<0.001
24-hour urine protein	-0.109	0.001
24-hour urine osmolality	0.952	<0.001
24-hour urine volume	0.578	<0.001
Serum creatinine	-0.198	<0.001
Serum cholesterol (total)	0.215	<0.001
Serum Triglyceride	0.001	0.925
Serum HDL	-0.729	<0.001
Serum LDL	0.289	<0.001
Hemoglobin	-0.427	<0.001

SUCOR=spot urine creatinine osmolality ratio, HDL=high density lipoprotein cholesterol, LDL=low density lipoprotein cholesterol

## Discussion

This study showed that the prevalence of low urine creatinine was 0.5% and high urine creatinine 6.4% in treatment-naïve HIV-positive subjects in Nigeria. Factors significantly associated with high urine creatinine were serum LDL, p=0.001 and serum HDL, p=0.028. There was very strong correlation between high urine creatinine and 24HUOsm (r=0.95), BMI, (r=0.74), CD4 cells count, (r=-0.71), serum HDL, (r=-0.73). In this study the prevalence of low urine creatinine was 0.5%. This does not agree with 8.1%t reported by Barr et al [14]. Similarly, the prevalence of high urine creatinine of 6.4% in our study was slightly higher than the 3.1% by Barr et al [14] in the same study, mentioned above. The observed difference might be explained by the differences in the study design. Our study was conducted in an HIV patient population in Nigeria, whereas theirs was in a general population in US. Some studies in Romania found a high prevalence of chronic kidney disease in HIV subjects on variable duration of antiretroviral therapy [15–17]. These studies assessed renal damage by estimated glomerular filtration rate, using MDRD equation that utilized serum creatinine. They did not, however, evaluate isolated urine creatinine in the study subjects.

We demonstrated, in this study, that high urine creatinine was significantly associated with serum LDL and serum HDL. From literature search we could not find any study that evaluated the direct influence of high urine creatinine on serum LDL or serum HDL. However, low serum HDL and low serum LDL characterize dyslipidemia found in chronic kidney disease. Proteinuria in renal disease induces the lipid synthesis by the liver. These are marked by triglyceride-rich apolipoprotein B (apoB)-containing complex lipoproteins, which have a significant atherogenic potential that may in turn adversely affect the kidney, and influence urine creatinine [18–20].

This study showed that there was very strong correlation between high urine creatinine and 24HUOSm, (r=0.95). A similar observation was reported in a study that assessed the utility of urine creatinine and urine osmolality in determining dilute or concentrated urine and the factors that influenced these. The study found that the magnitude of associations expressed as percent change was significantly stronger with creatinine than osmolality. It noted that urine osmolality, compared to urine creatinine, did not vary by diabetes status but was affected by daily total protein intake. Despite this association, the study observed that the feasibility of adopting urine osmolality adjustment and water intake recommendations before providing spot urine samples for environmental biomonitoring would merit further investigation.[12]

High urine creatinine has a high correlation with BMI, r=0.74, in this study. This is similar to the reports by Forbes et al [21] with r=0.99 and Baxmann et al [22] r=0.74. However, these two studies were carried out in a general population, unlike ours that was done in an HIV patient population. The slightly higher correlation seen in the Forbes et al [21] study above might have arisen from the fact that urine creatinine was evaluated in lean body mass, in subjects with underweight. In this study, it was shown that high urine creatinine has a high inverse correlation, r=-0.71, with CD4 cells count. Low CD4 cells count has been documented to be associated with underweight in HIV subjects [23]. This, perhaps, could explain the high but inverse correlation between high urine creatinine and CD4 cells count observed in our study. We also observed in our study that high urine creatinine has a high but inverse correlation, r=-0.73, with serum HDL. There was dearth of studies that assessed the relationship between serum HDL and high urine creatinine. This study showed that there was moderate correlation between high urine creatinine and 24HUV (r=0.58), and Hb (r=-0.43). Literature search did not reveal any study that evaluated the effects of urine volume or Hb on urine creatinine.

## Conclusion

The prevalence of low urine creatinine and high urine creatinine was low. Twenty four-hour urine osmolality. BMI, CD4 cells count and Hb were strong correlates of high urine creatinine. Lipid abnormalities were common in treatment-naïve HIV subjects with high urine creatinine. There is need for clinicians to routinely conduct urine creatinine and further search for abnormalities of serum lipids, weight changes, depressed immunity and anemia in HIV subjects with dilute or concentrated urine in the early stages of the infection. **Limitations:** Our study population was small. A larger study population would have been better, as it would have averted the skewed colinearity that voided the multivariate linear regression of urine creatinine with its potential risk factors. Assessment of CD4 cells count, staging of the HIV infection for all the subjects and concise definition of time from diagnosis of HIV infection to the conduct of this study were not done, but would have contributed in further defining the relationship between urine creatinine and these factors.

### What is known about this topic

- Urine creatinine is not commonly evaluated in routine clinical practice;
- Many environmental, physiologic and disease conditions affect urine creatinine;
- Some associated factors of dilute urine and concentrated urine have been identified; concentrated urine is associated with progression of chronic kidney disease.

### What this study adds

- The prevalence of dilute urine (0.5%)and concentrated urine (6.4%) are both low in treatment-naïve HIV-positive subjects in Nigeria;
- High urine creatinine was significantly associated with serum LDL and serum HDL in treatment-naïve HIV subjects;
- BMI and CD4 cells count have high correlation with high urine creatinine in treatment-naïve HIV subjects.
